# First report of *Meloidogyne naasi* parasitizing turfgrass in Portugal

**DOI:** 10.21307/jofnem-2020-088

**Published:** 2020-08-25

**Authors:** M. Clara Vieira dos Santos, M. Teresa M. Almeida, Sofia R. Costa

**Affiliations:** CBMA – Centre of Molecular and Environmental Biology, Department of Biology, University of Minho, Campus de Gualtar, 4710-057 Braga, Portugal

**Keywords:** Detection, Diagnosis, Iberian Peninsula, *Pasteuria penetrans*, Root-knot nematode, Worm-star

## Abstract

In an exploratory sampling of a football field in Porto, Portugal, the root-knot nematode, *Meloidogyne naasi*, previously unreported from the Iberian Peninsula, was detected. Diagnosis was based on the analysis of perineal patterns and esterase phenotypes of females excised from grass roots, morphometrics and molecular analysis (PCR with specific primers and analysis of partial 28S sequences obtained by amplification using the primers D2A/D3B) of second-stage juveniles (J2) extracted from soil. When collected in water, J2 aggregated into a worm-star. Endospores of *Pasteuria penetrans* were frequently found attached to the J2. To our knowledge, this is the first report of *M. naasi* in Portugal and in the Iberian Peninsula, and the first report of worm-star formation in *Meloidogyne.*

The barley root-knot nematode, *Meloidogyne naasi* Franklin, 1965 was originally described from field crops (cereals, grasses, and sugarbeet, *Beta vulgaris* L.) in England and Wales (Franklin, 1965). According to the EPPO Global Database, this nematode is present in temperate regions in all continents: Africa (Libya); Asia (Iran); Europe (Belgium, Czech Republic, Denmark, France, Germany, Hungary, Ireland, Italy, Malta, the Netherlands, Norway, Poland, Serbia and UK); North America (Canada, USA); Oceania (New Zealand) and South America (Argentina, Chile) (EPPO, 2020). To our knowledge, this is the first record of this nematode in Portugal and in the Iberian Peninsula.

A football field in Porto, Portugal, with poor turf performance caused by various plant health issues, including nematode parasitism, was sampled in June 2019. This field was originally a 60% *Lolium perenne* L. – 40% *Poa pratensis* L. mix, but had several patches of encroaching weed, *Poa annua*, and was scheduled for replacement. Twenty soil cores and their respective grass cover were collected and combined to form composite samples; the loose sandy soil was kept separate from the plug-like thick root masses of turfgrass.

Turfgrass roots were rinsed in water and upon observation with a stereomicroscope, small galls were detected, from which *Meloidogyne* females were excised (Fig. 1A). The perineal patterns from five adult females were prepared in 45% lactic acid and mounted in glycerin (Hartman and Sasser, 1985). Shape was round to ovoid with a low to medium-high round dorsal arch with coarse irregular striae and no or inconspicuous lateral lines. The most distinct characteristic was the presence of very distinct phasmids with the distance between them (14.1-20.0 µm, mean 18.1 µm) being only slightly smaller than the vulva width (17.9-22.4 µm, mean 20.7 µm) as described by Franklin (1965). A fold covering the anus was observed in three of the five perineal patterns. No striae were observed in the perivulval area (Fig. 1B).

**Figure 1:**
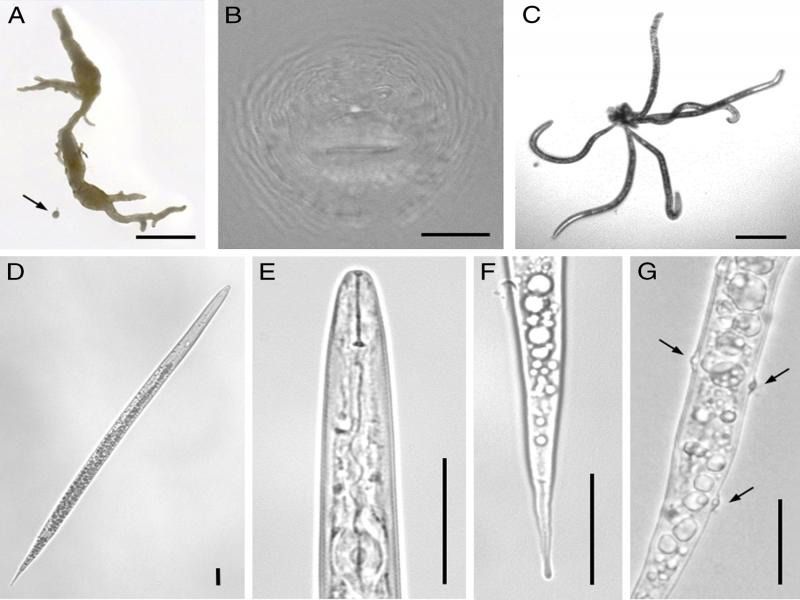
Root-knot nematodes (*Meloidogyne naasi* Franklin, 1965) extracted from soil and root samples collected from a football field in Portugal. A: turfgrass root showing galling by root-knot nematodes and excised *M. naasi* female (arrow); B: perineal pattern; C-G: second-stage juveniles: C: worm-star aggregation; D: whole body; E: pharyngeal region; F: tail; G: *Pasteuria penetrans* (ex Thorne, 1940) Sayre and Starr, 1985 endospores (arrows) attached to cuticle. Bars = 2000 µm (A); 125 µm (C); 25 µm (B, D-G).

Active soil nematodes were extracted from 100-ml subsamples by means of the tray method (Whitehead and Hemming, 1965). *Meloidogyne* second-stage juveniles (J2) detected in soil suspensions with an inverted microscope were picked and transferred to distilled water in staining glass blocks. After a few hours, these J2 formed ‘worm-star’ aggregates seemingly caused by attachment of several juveniles by their tails (Fig. 1C). In *Caenorhabditis elegans* (Maupas, 1900) Dougherty, 1955, this aggregation may be caused by a *Leucobacter* pathogen, that causes adhesion at the tail spikes within minutes of exposure, followed by bacterial proliferation in the host and death of all trapped nematodes in 24 to 28 hr (Hodgkin et al., 2013). Artificial exposure of *C. elegans* to other coryneform bacteria of the genus *Corynebacterium* has resulted in a similar aggregation, leading to nematode lysis and death after 2 to 4 days incubation (Antunes et al., 2016). Although we cannot deny or confirm the cause since it was not an objective of this investigation, we cannot exclude the action of a similar organism. To our knowledge, worm-star formation has not been previously reported on *Meloidogyne* or on any plant-parasitic nematode, although aggregation in ‘rosettes’ of entomopathogenic nematodes (Pye and Burman, 1981; Stock et al., 2005) and ‘medusa-heads’ or ‘sunflowers’ of human parasites (Yoeli, 1957) have been reported.

Seventeen J2 specimens were heat-killed, mounted in water in temporary slides, and photographed using a Leica DM 5000B + CTR 5000 microscope system (Fig. 1D-G). The J2 were slender and had pointy tails (Fig. 1D). Heads were round and not offset, with a delicate stylet (Fig. 1E). Tails were long, slightly enlarged, and often irregular at the tip (Fig. 1F). Endospores of *Pasteuria penetrans* (ex Thorne, 1940) Sayre and Starr, 1985, were frequently detected attached to the cuticle of J2 (Fig. 1G). Digital images were measured with ImageJ v. 1.52a (http://imagej.nih.gov/ij) to obtain J2 morphometric characters, presented in Table 1. Second-stage juveniles measured fresh in water were longer than those originally described by Franklin (1965) in lactophenol, but with a similar body length-maximum width proportion (*a*), and similar stylet size. Tails were longer than originally described, representing a larger part of the body, thus with a smaller *c* than described.

**Table 1.** Morphometric characters of second-stage juveniles in the Portuguese population of *Meloidogyne naasi* Franklin, 1965.

	*M. naasi* Portuguese population			*M. naasi* Original description (Franklin, 1965)		
Characters	*n*	Mean	Range	*n*	Mean	Range
Body length	17	457.3	440.6-487.2	25	435	418-465
Maximum body width	17	15.5	14.5-16.7	25	15	14-18
Stylet length	17	14.4	13.6-15.2	25	14	13-15
Tail length	13	82.8	76.4-89.9	25	70	52-78
Hyaline tail terminus	14	26.6	23.2-32.0	25	n.d.	n.d.
*a*	17	29.6	27.8-32.6	25	28	25-32
*c*	13	5.5	5.2-5.8	25	6.2	n.d.

**Notes:** All measurements in µm. a, c De Man indices; n.d. not determined.

Biochemical identification was performed by esterase phenotype analysis. Females were excised from infected roots of the turfgrass mix and transferred to sealed micro-hematocrit tubes with 5 μl of extraction buffer (20% sucrose with 1% Triton X-100) and macerated with a pestle. After centrifugation, the protein extracts were submitted to electrophoresis in polyacrylamide gels according to Pais and Abrantes (1989). Gels were stained for esterase activity using α-naphtyl acetate as substrate. Protein extracts of a *Meloidogyne javanica* (Treub, 1885) Chitwood, 1949 isolate were included in the gel as reference (Rm = 34.8, 39.3, 40). An individual V1F1 band (Rm = 45.5) esterase phenotype was detected (Esbenshade and Triantaphyllou, 1985).

For molecular analyses, DNA was extracted from 1, 5, or 10 J2 picked from soil suspensions into 10 μl of sterilized MilliQ water on a glass microscope slide. The nematodes were macerated with a pipette tip and collected in 20 μl sterilized MilliQ water and stored at −20°C (Harris et al., 1990). PCR with the species-specific primers N-ITS/R195 was performed as described in Zijlstra et al. (2004). DNA extracts from J2 of a *Meloidogyne incognita* (Kofoid and White, 1919) Chitwood, 1949 isolate were included as a negative control. An expected PCR product of ca. 430 bp was obtained and no amplification was detected for the *M. incognita* DNA. To confirm the identification, partial 28S sequences were amplified with the primers D2A/D3B designed by Nunn (1992) as described in Ye et al. (2015). The obtained PCR fragments (ca. 980 bp) were purified and sequenced by Sanger sequencing at STAB VIDA (Caparica, Portugal) and deposited in GenBank (accession numbers MT478996-MT478999). The sequences showed more than 99% similarity to the *M. naasi* 28S ribosomal RNA gene partial sequence available in GenBank.

*Meloidogyne naasi* is one of the most important root-knot nematodes affecting grains in European countries and has also been detected in fields where potatoes (*Solanum tuberosum* L.) are grown (Zijlstra et al., 2004; Wesemael et al., 2011). The potential impact of *M. naasi* on turfgrass and crop fields and the growing need for effective and sustainable management programs reinforces the importance of its monitoring and the need to assess its distribution and potential dissemination. An accurate diagnosis is also essential to support decision-making on quarantine of imported/exported plant materials and must include morphometric, biochemical, and molecular data (Hunt and Handoo, 2009). Finally, if the worm-star aggregation reported here can be attributed to a *Leucobacter* bacterial pathogen, this may open new research possibilities for the biological control of root-knot nematodes.
